# An unusual delayed complication of paraffin self-injection for penile girth augmentation

**DOI:** 10.1186/1471-2490-13-66

**Published:** 2013-12-01

**Authors:** Mario De Siati, Oscar Selvaggio, Giuseppe Di Fino, Giuseppe Liuzzi, Paolo Massenio, Francesca Sanguedolce, Giuseppe Carrieri, Luigi Cormio

**Affiliations:** 1 Department of Urology and Renal Transplantation, University of Foggia, Viale Pinto n° 1, 71122, Foggia, Italy; 2 Department of Pathology, University of Foggia, Viale Pinto n° 1, 71122, Foggia, Italy

**Keywords:** Lipogranuloma, Penis, Male genitalia, Mineral oils

## Abstract

**Background:**

Penile self-injection of various oils is still carried out among Eastern Europe people for penile girth augmentation despite the potential destructive complications of this practice are well known. Penile reactions to such foreign bodies include scarring, abscess formation, ulceration, and even Fournier’s gangrene; voiding problems due to mineral oil self-injection have been reported only once. To our knowledge, we describe the first case of paraffin self-injection for penile girth augmentation presenting with acute urinary retention.

**Case presentation:**

A 27-year-old Romanian man presented with severe penile pain and acute urinary retention five years after having practiced repeated penile self-injections of paraffin for penile girth augmentation. The penile shaft was massively enlarged, fibrotic and phymotic; urethral catheterization failed due to severe stricture of the proximal pendulum urethra. The patients refused placement of a suprapubic catheter and underwent immediate penile surgical exploration. The scarred tissue between dartos and Buck’s fascia and a fibrotic ring occluding the urethra were removed and the penile skin reconstructed. Pathology confirmed the diagnosis of paraffinoma. The patient resumed normal voiding immediately after catheter removal on second postoperative day; he was very pleased with cosmetic, sexual and voiding results at six weeks, six months and 1 year follow-up.

**Conclusions:**

The present report describes a novel complication of penile self-injection for penile girth augmentation. Because of the increasing number of patients seeking penile augmentation, physicians dealing with sexual medicine should pay more attention to such request to prevent the use of non medical treatments that can turn into medical disasters.

## Background

Penile girth augmentation (PGA) by means of subcutaneous injection of various oils is still carried out among people from the Eastern Europe despite the potential destructive complications of this practice are well known since the early 1900s [1]. As a matter of fact, several kinds of foreign body reactions, including penile scarring and deformity, abscess formation, ulceration, erectile dysfunction and even Fournier’s gangrene, have been reported following injection of these oils [1-12]. Reactions to cod fish oil tend to occur shortly (1–2 weeks) after injection [13], whereas reactions to paraffin or mineral oil tend to occur 1 to 2 years after injection [1]; both usually cause skin scarring leading to paraphimosis and penile deformity, or skin infection leading to purulent discharge, ulceration and even necrosis. Voiding problems have previously been reported only once, in a 64-year-old man with a 9-cm firm irregular penile mass after repeated self-injections on mineral oil [2]. Herein we report the first case, to our knowledge, of penile paraffinoma presenting with acute urinary retention five years after repeated penile self-injection of paraffin for PGA.

## Case presentation

**Figure 1:**
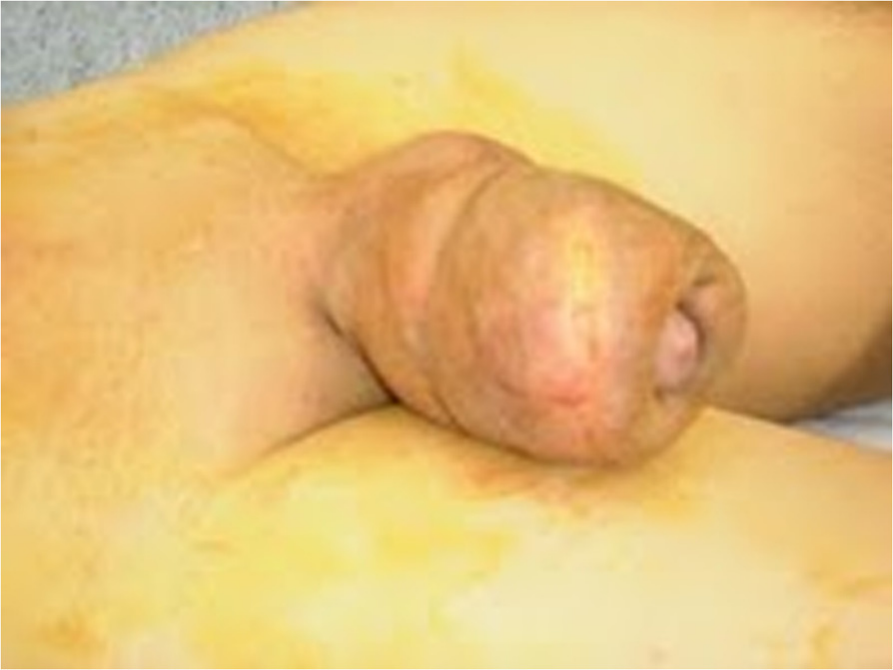
**Massively enlarged and deformed penile shaft with phymotic foreskin.** Physical examination revealed a penile shaft enlarged, deformed and fibrotic, with phymotic foreskin; the scrotum was normal.

**Figure 2:**
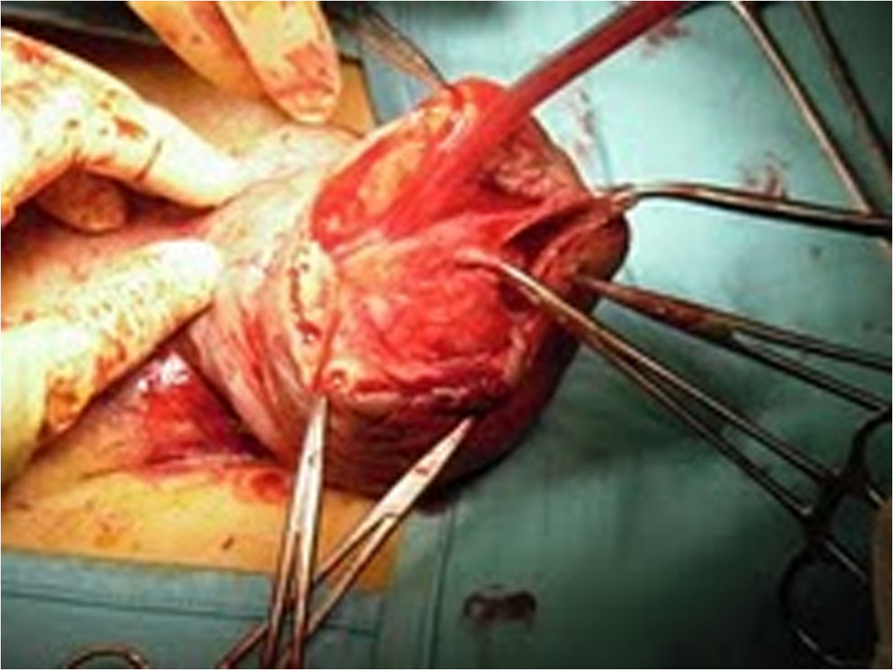
**Midline dorsal penile shaft incision.** The operation started with a midline dorsal penile shaft incision to access and remove the scarred tissue between dartos and Buck’s fascia dorsally.

**Figure 3:**
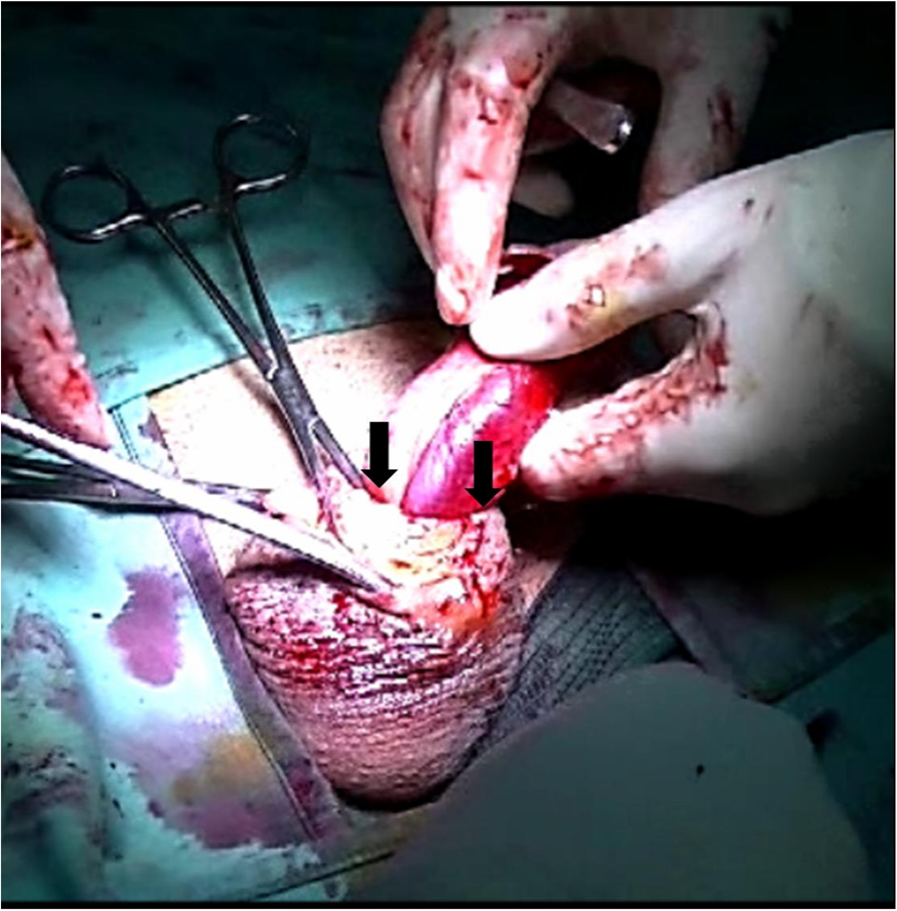
**Fibrotic ring occluding the urethra.** Following complete subcoronal incision and midline ventral penile shaft incision, the scarred tissue of the ventral penile shaft, including a fibrotic ring occluding the urethra, was accessed and removed.

A 27-year-old Romanian man presented with severe penile pain and acute urinary retention. He had an unremarkable medical history. On physical examination, the penis was massively enlarged and the foreskin phymotic while the scrotum was normal (Figure 1). He reported having practiced, approximately 5 years before, five penile self-injections of paraffin for PGA and having had, following each injection, an immediate inflammatory reaction that ceased spontaneously in a few days. The four years after the injections had been uneventful, whereas in the fifth year he noticed progressive penile swelling with increasing intercourse and voiding difficulties up to the present episode of urinary retention. Urethral catheterization failed due to severe stricture of the proximal pendulum urethra. The patients refused placement of a suprapubic catheter; therefore, penile surgical exploration was immediately carried out. Following midline dorsal penile shaft incision (Figure 2), the scarred tissue between dartos and Buck’s fascia was widely excised. Then we carried out a complete subcoronal and a midline ventral penile shaft incision to free the ventral penile portion from the scarred tissue. In this phase, a fibrotic ring occluding the urethra was encountered and removed (Figure 3). The penile incisions were finally closed and a detensioning prepubic skin plasty (transverse incision and longitudinal suture) was carried out to prevent a buried penis effect.

**Figure 4:**
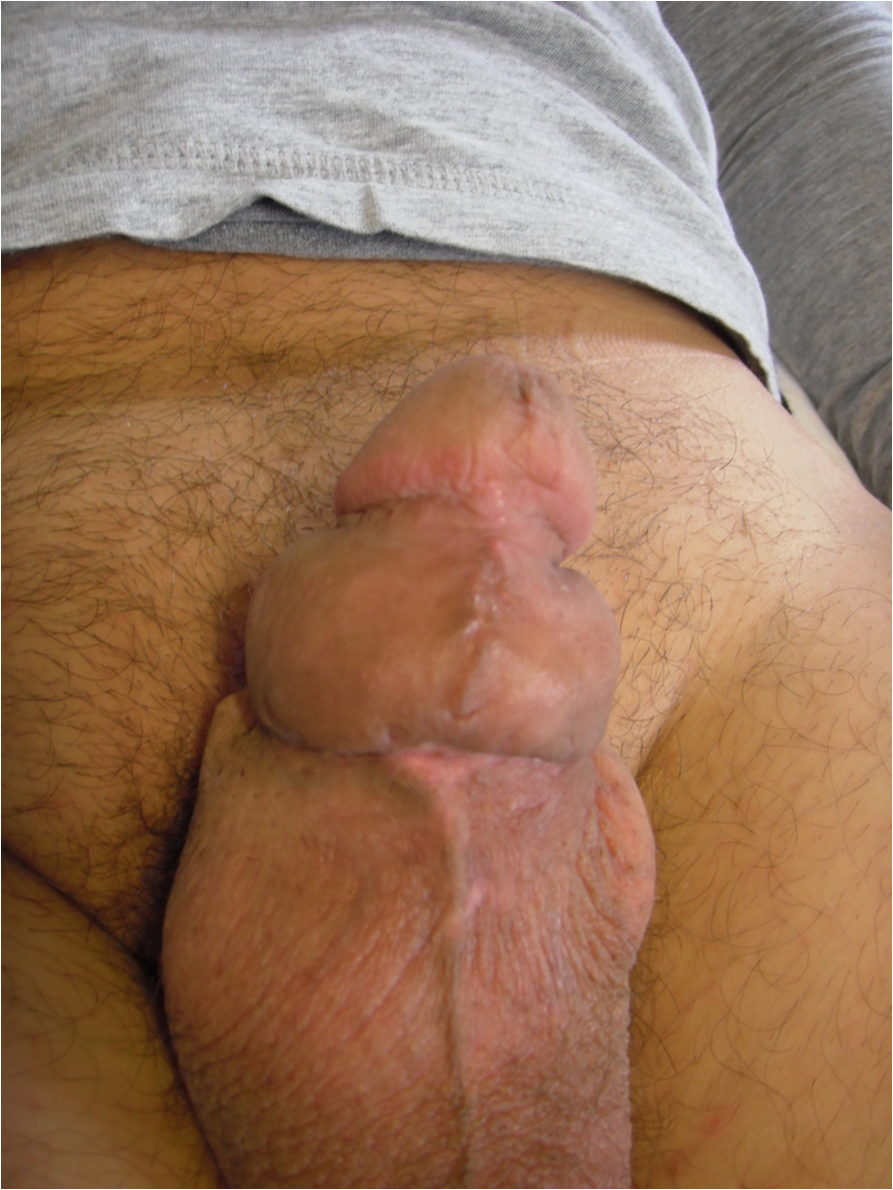
**Cosmetic results six weeks after surgery.** Six weeks after surgery, the sutured had healed well and the patient was satisfied with cosmetic and functional results.

The postoperative course was uneventful; the urethral catheter was removed on second postoperative day and the patient discharged 24 hours later, after a peak flow rate of 25 mL/sec and absence of post-void residual urine having been demonstrated by uroflowmetry and bladder ultrasounds. Histological examination confirmed the diagnosis of paraffinoma, showing a foreign-body type chronic granulomatous inflammation and epithelioid giant cells. Six weeks after surgery the patient reported being satisfied with the cosmetic result (Figure 4) as well as with his sexual and voiding functions; uroflowmetry showed a peak flow rate of 26 mL/sec and there was no post-void residual urine at bladder ultrasounds. At 6- and 12- months follow-up, he continued to be very pleased with cosmetic, sexual and voiding results.

## Conclusions

The number of patients seeking penile augmentation is continuously increasing. The vast majority of them has a normally sized and normally functioning penis but is dissatisfied by the girth. A recent study on healthy young Korean military men [14] pointed out that 24% of them underestimated their penile size. It is therefore intuitive that some of them could look for PGA.

PGA can be achieved by longitudinally grafting the tunica albuginea or by subcutaneous injection of filling substances. While grafting requires surgery, subcutaneous injection is easily carried out by medical and even non-medical personnel, thus making such procedure particularly attractive.

Filling substances used in the non-medical setting include paraffin [1], vaseline [10], mineral oil [2], cod liver oil [13], metallic mercury [3], and petroleum jelly [8]; they all could cause a foreign body reaction leading to penile scarring and deformity, abscess formation, ulceration, erectile dysfunction and even Fournier’s gangrene [1-12]. The foreign body reaction usually involves the penile skin and the dartoic fascia; conversely, involvement of tissues under Buck’s fascia is extremely rare. Reviewing the literature we found only one case of corpus cavernosum involvement [15] and one case of urethral involvement [2] leading to voiding difficulties but not urinary retention. Therefore, the present is, to our knowledge, the first reported case of urinary retention following repeated paraffin self-injections for PGA.

There are two interesting features of the reported case. The first is the delayed occurrence of the voiding problems. As mentioned above, delayed reactions tend to occur between first and second year after penile paraffin injection [1], whereas in our patient the delayed reaction occurred after approximately five years. This finding suggests that the chronic inflammatory reaction to such foreign body could theoretically reactivate at later stages and that such delayed inflammatory process is more likely to move towards the underlying tissues rather than the stabilized overlying penile skin. The second is the sudden resolution of the voiding problems after surgery. As a matter of fact, complete removal of the sclerosing lipogranuloma compressing the corpus spongiosum up to occluding the urethral lumen resulted in prompt resumal of spontaneous micturition with a normal flow rate and absence of post-void residual urine volume.

In conclusion, urologists tend to be quite indifferent to their patients’ complaints about penile girth, often proposing psychiatric consultations rather than surgical solutions. Such indifference, together with the availability of non medical treatments administered by non-medical personnel and popularized on the web, is likely responsible for patients continuing to adopt non-medical solutions in spite of their well-known risks. Physicians dealing with sexual medicine should provide more attention to these patients and search for simple surgical solutions, thus avoiding non-medical solutions that can turn into medical disasters.

## Consent

Written informed consent was obtained from the patient for publication of this Case report and any accompanying images. A copy of the written consent is available for review by the Editor of this journal.

## Abbreviations

PGA: Penile girth augmentation.

## Competing interests

The authors declare that they have no competing interests.

## Authors’ contributions

MD, manuscript conception. OS, manuscript drafting. GD, manuscript drafting. GL, data acquisition. PM, data acquisition. FS, pathology support. GC, supervision. LC, supervision. All authors read and approved the final manuscript.

## Pre-publication history

The pre-publication history for this paper can be accessed here:

http://www.biomedcentral.com/1471-2490/13/66/prepub
